# The role of glycogen synthase kinase 3 beta in neurodegenerative diseases

**DOI:** 10.3389/fnmol.2023.1209703

**Published:** 2023-09-15

**Authors:** Honglu Yu, Min Xiong, Zhentao Zhang

**Affiliations:** ^1^Department of Neurology, Renmin Hospital of Wuhan University, Wuhan, China; ^2^TaiKang Center for Life and Medical Sciences, Wuhan University, Wuhan, China

**Keywords:** protein aggregation, Alzheimer’s disease, Parkinson’s disease, Huntington’s disease, amyotrophic lateral sclerosis

## Abstract

Neurodegenerative diseases (NDDs) pose an increasingly prevalent threat to the well-being and survival of elderly individuals worldwide. NDDs include Alzheimer’s disease (AD), Parkinson’s disease (PD), Huntington’s disease (HD), amyotrophic lateral sclerosis (ALS), and so on. They are characterized by progressive loss or dysfunction of neurons in the central or peripheral nervous system and share several cellular and molecular mechanisms, including protein aggregation, mitochondrial dysfunction, gene mutations, and chronic neuroinflammation. Glycogen synthase kinase-3 beta (GSK-3β) is a serine/threonine kinase that is believed to play a pivotal role in the pathogenesis of NDDs. Here we summarize the structure and physiological functions of GSK3β and explore its involvement in NDDs. We also discussed its potential as a therapeutic target.

## 1. Introduction

Neurodegenerative diseases (NDDs) are characterized by the progressive loss of neurons in the nervous system. These diseases typically cause damage to the brain and spinal cord, which in turn affects various physiological functions such as cognition, movement, perception, the autonomic nervous system, and even breathing and circulation (Dugger and Dickson, 2017). NDDs include Alzheimer’s disease (AD), Parkinson’s disease (PD), Huntington’s disease (HD), and Amyotrophic lateral sclerosis (ALS), among others (Boland et al., 2018). Symptoms of these conditions worsen over time. Treatment focuses on improving patients’ quality of life by alleviating symptoms, but there is currently no effective cure. Increasing evidence suggests that changes occur in the activity and levels of GSK-3β in NDDs, and the GSK-3β signaling pathway is now recognized as a key signaling pathway promoting neurodegeneration. Inhibition of GSK-3β has emerged as a promising therapeutic approach for NDDs (Rippin and Eldar-Finkelman, 2021).

## 2. Structure and biological function of GSK3β

**Figure 1:**
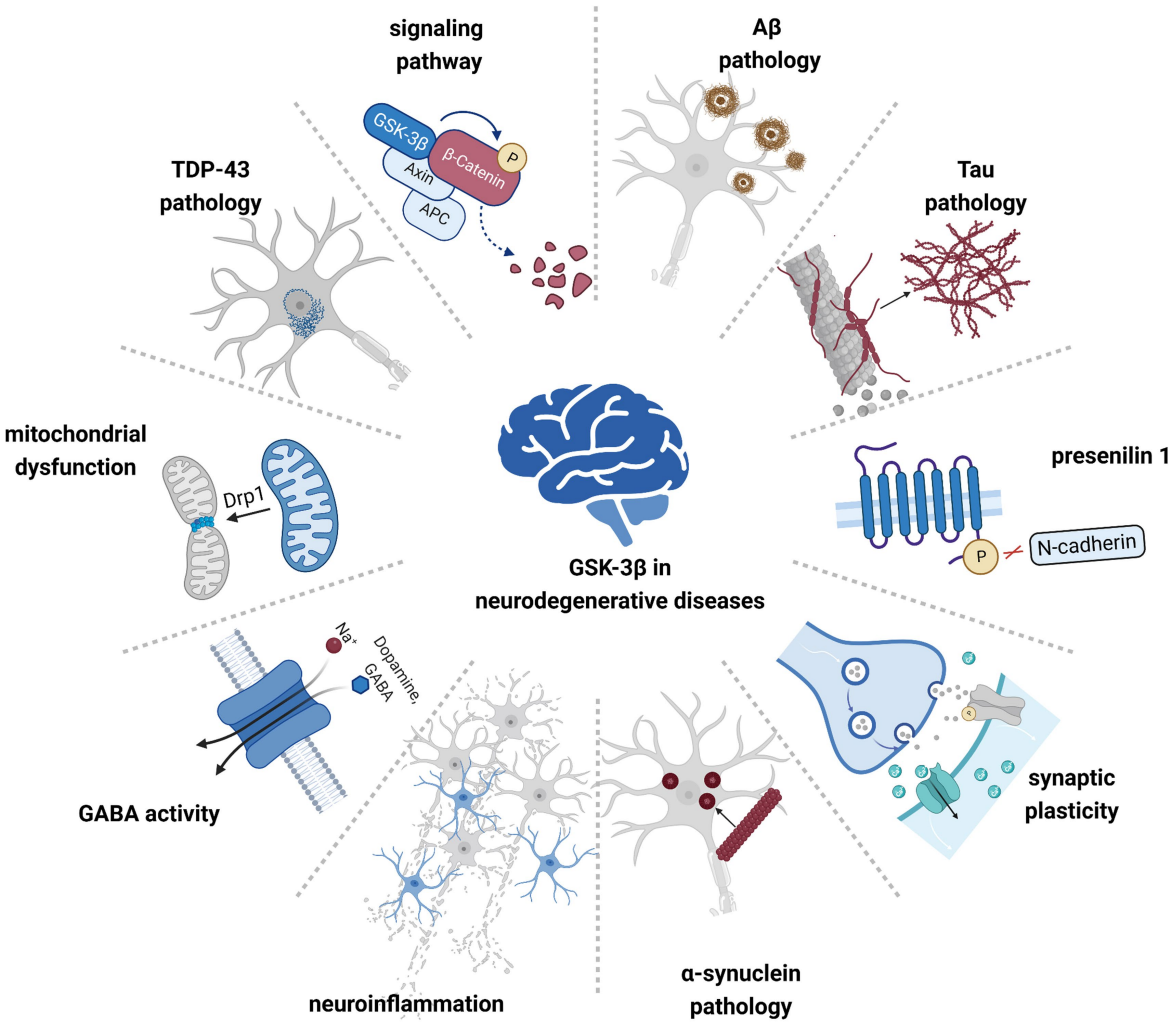
GSK-3β is involved in the pathogenesis of multiple neurodegenerative diseases. Hyperactivated GSK-3β contributes to pathological protein aggregation, neuroinflammation, and mitochondrial dysfunction, affecting cellular signaling pathways and neuronal synaptic plasticity.

Glycogen synthase kinase-3 (GSK-3) was initially discovered for its ability to phosphorylate glycogen synthase and regulate glucose metabolism in response to insulin. Subsequent studies have shown that it is a widely expressed serine/threonine kinase that can phosphorylate over 100 protein substrates and is located at the intersection of many signaling pathways (Beurel et al., 2015). Encoded by two different genes located on chromosomes 19 and 3, GSK-3 has two unique isoforms: GSK-3α (51 kDa) and GSK-3β (47 kDa). These isoforms share a striking 97% amino acid sequence identity within the catalytic domain and 84% overall amino acid sequence similarity, suggesting potential common biological functions. For example, they regulate cell signaling, participate in biological metabolism, cell growth, proliferation, and differentiation, and are critical regulatory factors in many neurodevelopmental processes (Marosi et al., 2022). GSK-3β exhibits high levels of expression in the central nervous system (CNS; Yao et al., 2002). The kinase activity of GSK-3β is regulated by phosphorylation. Within the cell, serine kinases such as protein kinase B (PKB/AKT) and AMP-activated protein kinase (AMPK) target the 9th serine residue (Ser9) of GSK3β, resulting in its phosphorylation (pGSK-3β-Ser9) and subsequent inactivation. In contrast, tyrosine kinases can phosphorylate the 216th tyrosine residue (Tyr216) of GSK-3β (resulting in pGSK-3β-Tyr216), leading to a fivefold increase in its enzymatic activity. GSK-3β interacts with numerous molecules intricately associated with NDDs, including hippocampal cell proliferation, neuronal development and regeneration, cell cycle regulation, and neuronal polarization (Pardo et al., 2016; Demuro et al., 2021; see Figure 1).

## 3. GSK-3β in AD

AD is the most common neurodegenerative disease, encompassing 60–80% of global dementia cases (Gauthier et al., 2022). Cognitive and non-cognitive symptoms are prominent clinical features. Early stages manifest with challenges in recalling recent discussions, names, events, emotional responses, or even depressive symptoms, which evolved into communication impairments, confusion, compromised decision-making, and behavioral alterations. As the disease advances, walking difficulties, speech impairments, and swallowing problems become prevalent (Breijyeh and Karaman, 2020). Regarding genetic characteristics, Alzheimer’s disease (AD) can be categorized into familial and sporadic forms. Familial AD is associated with mutations in three genes: APP, PSEN1, and PSEN2. Sporadic AD is the most prevalent form (Uddin et al., 2021). It has prompted various hypotheses elucidating its onset, encompassing the cholinergic, amyloid, Tau protein proliferation, mitochondrial cascade, inflammation, and neurovascular hypotheses. The pathological features include the formation of senile plaques composed of amyloid-β (Aβ) and intracellular neurofibrillary tangles (NFTs) composed of hyperphosphorylated Tau (Penke et al., 2019; Kent et al., 2020). This aggregation of Aβ and Tau detrimentally affects synaptic plasticity and triggers neuronal cell demise (Scheltens et al., 2021). Elevated GSK-3β activity has been observed in the brains of AD patients and various AD mouse models. Upregulation of GSK-3β has been shown to instigate AD pathology, cognitive decline, and glial cell proliferation (Albeely et al., 2022; Chauhan et al., 2022).

### 3.1. GSK-3β regulates Aβ pathology

In the context of Alzheimer’s disease (AD), the accumulation of Aβ protein precedes the formation of paired helical filaments (PHFs), which constitute the neurofibrillary tangles (NFTs). The principal events underlying AD involve the aberrant metabolism of amyloid precursor protein (APP) and subsequent Aβ deposition. The processing of APP occurs through either the non-amyloidogenic or the amyloidogenic pathway. Sequential cleavage of APP by α-secretase and γ-secretase generates three fragments: the secreted C-terminal fragment (sAPPα), p3, and the APP intracellular domain (AICD) (Aguzzi and O'Connor, 2010). The alternative pathway includes cleavage by β-secretase, also known as β-site APP cleaving enzyme (BACE-1), followed by cleavage by γ-secretase, which produces Aβ (Dimitrov et al., 2013). Normally, APP cleavage predominantly follows the non-amyloidogenic pathway. Cleavage of APP via the amyloidogenic pathway leads to the production of Aβ. β-Secretase (BACE1) is the rate-limiting enzyme that generates Aβ in the amyloidogenic pathway (Bahn et al., 2019). GSK-3β intensifies the activity of β-secretase (BACE1) and mediates the toxicity of Aβ aggregates. Inhibition of GSK-3β diminishes BACE1-mediated APP cleavage, thus reducing Aβ generation (Ly et al., 2013). Aβ aggregates activate microglia and trigger inflammation, leading to neurodegeneration and cell death (Wang et al., 2023).

### 3.2. GSK-3β regulates tau pathology

Tau is predominantly expressed in the axons of neurons and functions as a microtubule-associated protein. It possesses 45 phosphorylation sites and 79 serine and threonine residues available for phosphorylation (Duan et al., 2017). Abnormal phosphorylation of Tau protein leads to the formation of aggregates known as neurofibrillary tangles (NFTs; Sayas and Ávila, 2021). One of the primary kinases responsible for Tau phosphorylation is GSK-3β, which phosphorylates numerous serine or threonine residues on Tau. The aggregation of hyperphosphorylated Tau represents an additional pathological hallmark of AD (Muralidar et al., 2020). This aggregation of Tau is directly linked to the upregulation of GSK-3β, further intensifying Tau pathology in murine brains. Conversely, deletion of GSK-3β attenuates Tau phosphorylation, hippocampal degeneration, and learning defects in mouse models of AD. Inhibition of GSK-3β reduces Tau phosphorylation and neurodegeneration (Hurtado et al., 2012; Sayas and Ávila, 2021).

### 3.3. GSK-3β regulates the function of presenilin 1

Presenilin 1 (PS1), a product of the PSEN1 gene, has been verified as an important causative factor for familial AD (FAD). PS1 is a transmembrane protein with nine domains linked by hydrophilic loops in the extracellular area or cytosol. It plays a role in cleaving APP and also affects other processes like Notch signaling, β-cadherin processing, and calcium metabolism (Giau et al., 2019). PS1 functions in the C-terminal transmembrane region of APP, directing the generation of amyloid-β peptide (Aβ42) through γ-secretase after α- and β-secretase cleavage (Bagaria et al., 2022). GSK-3β phosphorylates PS1 at serine residues (Ser353 and Ser357), restraining APP cleavage and Aβ production while modulating the Aβ 42/40 ratio, thereby exacerbating AD (Maesako et al., 2011). Moreover, PS1 forms a synaptic trimeric complex with N-cadherin/β-catenin, facilitated by its ring structure domain. GSK-3β-mediated PS1 phosphorylation also affects its interaction with N-cadherin, disrupting their binding and leading to synaptic and neuronal activity deficits (Uemura et al., 2007). This intricate interplay among PS1, GSK-3β, and their impact on Aβ metabolism and synaptic function contributes significantly to the pathogenesis of Alzheimer’s disease.

### 3.4. GSK-3β regulates synaptic plasticity

Synaptic dysfunction is an early sign of AD (Tönnies and Trushina, 2017). Long-term potentiation (LTP) and long-term depression (LTD) are crucial for regulating synaptic connections between neurons (Warpechowski et al., 2023). LTP refers to the long-lasting increase in synaptic strength, while LTD is defined as the opposing process. In LTD, N-methyl-D-aspartate (NMDA) receptor channels and alpha-amino-3-hydroxy-5-methyl-4-isoxazolepropionic acid (AMPA) receptor channels on the postsynaptic membrane play a critical role. AMPA is a sodium-potassium cation channel that helps activate neurons, while NMDA is a calcium ion channel. High-frequency stimulation leads to a high influx of calcium ions, which then bind with calmodulin (CaM) to form Ca^2+^/CaM complexes. These complexes activate calcium/calmodulin-dependent protein kinase II (CaMKII), thereby inducing LTP. LTP is involved in synaptic formation, plasticity, learning, memory, as well as excitotoxicity (Vyklicky et al., 2014). Low-frequency electrical stimulation causes a minor influx of calcium ions, leading to AMPA phosphorylation and LTD (Collingridge and Monaghan, 2022). GSK-3β is widely expressed in the hippocampus, a key brain region involved in learning and memory, where it plays a crucial regulatory role in balancing LTP and LTD. Inducing LTP can prevent the occurrence of LTD, and the induction of LTD is associated with a decrease in the phosphorylation of GSK-3β at Ser9. Inhibiting the active form of GSK-3β is beneficial for the induction of LTP (Peineau et al., 2007). Transgenic mice overexpressing the active form of GSK-3β display reduced LTP and abnormalities in hippocampus-dependent spatial and fear memory formation. Defective LTP can be alleviated or rescued by the long-term administration of the GSK-3β inhibitor lithium (Hooper et al., 2007). Therefore, GSK-3β plays a critical role in initiating NMDA-induced LTD in neurons.

## 4. GSK-3β in PD

Parkinson’s disease is a progressive neurodegenerative disorder with significant morbidity and mortality (Halli-Tierney et al., 2020). The pathological hallmark of PD is the progressive loss of dopaminergic neurons in the substantia nigra pars compacta (SNpc) and the accumulation of misfolded α-synuclein (Armstrong and Okun, 2020). Cardinal motor symptoms such as tremors, rigidity, bradykinesia, and postural instability are typically observed. PD can also present with non-motor symptoms (NMSs), including sleep disorders, constipation, urinary dysfunction, orthostatic hypotension, cognitive decline, depression, and anxiety (Schapira et al., 2017). Multiple pathways and mechanisms are involved in the molecular pathogenesis of PD, including α-synuclein deposition, oxidative stress, mitochondrial function, calcium homeostasis, axonal transport, and neuroinflammation (Balestrino and Schapira, 2020). Both environmental and genetic factors have been found to contribute to the development of PD (Yuan et al., 2022). Notably, the activity of GSK-3β is elevated in the striatum of PD patients compared to controls, indicating the involvement of GSK-3β in the pathogenesis of PD.

### 4.1. GSK-3β regulates α-synuclein pathology

α-Synuclein is a neuronal protein primarily located at the presynaptic terminal, and it is the major proteinaceous component of Lewy bodies (Lashuel, 2020). α-Synuclein plays a role in regulating synaptic activity and neurotransmitter release (Burré, 2015). During the onset of PD, soluble monomers of α-synuclein form oligomers, which then aggregate to form insoluble fibrils. Mutations or overexpression of α-synuclein promote its aggregation (Burré et al., 2018). Increasing evidence suggests that GSK-3β may play a crucial role in the expression and aggregation of α-synuclein. Both active and inactive forms of GSK-3β colocalize with pathologic α-synuclein in Lewy bodies in postmortem examinations of PD patient (Nagao and Hayashi, 2009). Transgenic mice overexpressing α-synuclein demonstrate heightened GSK-3β activity (Duka et al., 2009). Inhibition of GSK-3β can reduce the expression of α-synuclein and prevent cell death in cellular models of PD, suggesting that inhibiting GSK-3β may have neuroprotective effects on dopaminergic neurons by reducing the toxicity of α-synuclein overexpression. These findings emphasize the pivotal role of GSK-3β in α-synuclein pathology.

### 4.2. GSK-3β regulates dopamine receptor signaling

Dopamine is a pivotal neurotransmitter within the central nervous system, intricately regulating various functions such as motor control, cognition, emotion, and reward. It plays a critical role in the pathogenesis of PD (Latif et al., 2021). The PI3K/Akt pathway serves as the upstream regulatory factor of GSK-3β. The PI3K/Akt pathway is integral to dopamine receptor signaling, playing a pivotal role in various cellular processes, including apoptosis, transcription, and cell proliferation. Dysregulation of this pathway is associated with PD pathology (Timmons et al., 2009). In the dopaminergic neurons of individuals with PD, there is a reduction in the phospho-Akt/total Akt ratio (Malagelada et al., 2008). Specific PD medications activate the PI3K/Akt pathway, protecting dopaminergic neurons in the substantia nigra (Lim et al., 2008; Zhang et al., 2011). The Akt/GSK-3 cascade also intersects with dopamine D2 receptor effect (Gomez-Sintes et al., 2014). Dopamine D2 receptor activation triggers a signaling complex (PP2A, β-arrestin-2, and Akt) (Beaulieu et al., 2007). This complex leads to Akt dephosphorylation and subsequent activation of GSK-3β. When dopamine is present in excess, the activation of striatal GSK-3β depends on D2 receptor activity. This interaction underscores the involvement of GSK-3β signaling in dopamine-mediated locomotor activity. In PD models, inhibition of GSK-3β safeguards dopaminergic neurons from stress-induced damage, subsequently reducing dopamine-driven locomotor deficit (Beaulieu et al., 2004; Wang et al., 2007). These findings underscore the crucial role of the Akt/GSK-3β cascade as a significant signaling pathway influenced by dopamine dysregulation.

## 5. GSK-3β in HD

HD is a rare autosomal dominant genetic disorder resulting from the abnormal expansion of CAG trinucleotide repeats in the Huntingtin gene on the chromosome (Tabrizi et al., 2020). This expansion triggers the toxic form of the Huntingtin protein, leading to synaptic impairment, mitochondrial dysfunction, and disrupted axonal transport, ultimately contributing to motor, cognitive, and psychiatric impairments (Baake et al., 2017; Ghosh and Tabrizi, 2018). Emerging research also suggests that HD may be classified as a secondary tauopathy, with tau insoluble aggregates observed in late-stage HD (Salem and Cicchetti, 2023). Furthermore, there is increased activity of pGSK-3β-Tyr216 in the hippocampus of HD patients and mice, underscoring the potential significance of abnormal GSK-3β signaling in HD progression (L'Episcopo et al., 2016).

### 5.1. GSK-3β regulates tau pathology and neuronal degeneration in HD

Recent findings indicate that Tau undergoes pathological changes in HD, encompassing insoluble aggregate formation, altered levels, mis-splicing, hyperphosphorylation, and truncation within the brain (Gratuze et al., 2016; St-Amour et al., 2018; Masnata et al., 2020; Petry et al., 2023). Hyperphosphorylated Tau aggregates are observed in postmortem HD brain neurons within cortical, striatal, and hippocampal regions, coinciding with hippocampal neuronal loss (Fernández-Nogales et al., 2014; Vuono et al., 2015). Significantly, Tau knock-down has been demonstrated to attenuate motor abnormalities in an HD mouse model (Fernández-Nogales et al., 2014). This suggests the potential involvement of hyperphosphorylated Tau in HD’s behavioral and pathological aspects. The association between the mutation in the huntingtin gene and Tau dysregulation in HD remains unclear. Several hypotheses propose that the mutant huntingtin does not directly interact with Tau (Fernández-Nogales et al., 2014). Instead, it interacts with Tau kinases, phosphatases, and proteins engaged in Tau alternative splicing. Notably, GSK-3β and its active form (pGSK-3β-Tyr216) are elevated in the hippocampus of postmortem HD brain (L'Episcopo et al., 2016). Researchers have examined different Tau kinases and phosphatases in HD mouse models to unveil mechanisms underlying Tau hyperphosphorylation (Blum et al., 2015; Gratuze et al., 2015). They observed heightened levels of GSK-3β and its active form (pGSK-3β-Tyr216) in neurons and astrocytes of HD brains. Meanwhile, the localization of GSK-3β and its active form shifted from the cytoplasm to the nucleus in both neurons and astrocytes (L'Episcopo et al., 2016). This shift in localization from the cytoplasm to the nucleus suggests that GSK-3β may not only be heightened at the protein level but also upregulated in gene transcription in HD, ultimately contributing to excessive Tau phosphorylation (L'Episcopo et al., 2016). Furthermore, as HD progresses, levels of pGSK-3-Tyr216 and pTau steadily increase in the hippocampus, highlighting GSK-3β’s role in facilitating Tau hyperphosphorylation and pathological accumulation in HD.

### 5.2. GSK-3β regulates GABA activity

Both neurons and astrocytes are capable of synthesizing and releasing gamma-aminobutyric acid (GABA). The balance between excitatory glutamatergic and inhibitory GABAergic systems is crucial for motor and behavioral control (Koch and Raymond, 2019). One of the mechanisms underlying the development of HD is the disruption of GABAergic neurotransmission (Garret et al., 2018). Dysfunction in this circuitry may lead to the development of HD symptoms. Studies on postmortem brain tissue from HD patients have shown that GABA content is lower in the tail of the caudate nucleus, putamen, and cortex compared to non-HD controls. GABA typically suppresses dopamine release by activating GABA receptors on the soma and terminals of nigrostriatal neuron (Tyagarajan et al., 2011). Increased initial dopamine release or elevated extracellular glutamate levels may induce excitotoxicity, loss of cortical and striatal neurons, and loss of dopaminergic terminals, leading to motor disorder (Koch and Raymond, 2019). There is a unique phosphorylation site, Ser270, on the GABAergic postsynaptic scaffold protein Gephyrin, and phosphorylation of Gephyrin by GSK-3β reduces GABAergic transmission (Tyagarajan et al., 2011). Blocking Ser270 phosphorylation increases the density of Gephyrin clusters and the frequency of GABAergic postsynaptic currents in cultured hippocampal neurons (Rippin et al., 2021).

## 6. GSK-3β in ALS

ALS, also known as Lou Gehrig’s disease, is a neurodegenerative disorder that primarily affects motor neurons in the spinal cord, brainstem, and motor cortex, resulting in the gradual weakening of the muscles and eventually respiratory failure. The cause of ALS is unknown, with suggested involvement of signaling pathway alterations and factors such as protein misfolding and misassembly, mitochondrial dysfunction, oxidative stress, and neuroinflammation (Sever et al., 2022). ALS is divided into two types, with approximately 90–95% being sporadic cases (sALS) and the remaining 5–10% being familial cases (fALS) (Prasad et al., 2019). Research has found an increase in the levels of GSK-3β and phosphorylated GSK-3β at Tyr216 in the frontal cortex and hippocampus of ALS patients (Yang et al., 2008). GSK-3β is believed to affect the superoxide dismutase SOD1 gene and TAR DNA binding protein 43 (TDP-43), thus promoting the progression of ALS.

### 6.1. GSK-3β regulates TDP-43 pathology

TDP-43 is a 43 kDa protein encoded by the TARDBP gene that binds to DNA and RNA. It plays a crucial role in regulating RNA metabolism, axonal transport, vesicular trafficking, and stress response mechanisms (Palomo et al., 2019). Hyperphosphorylated and ubiquitinated TDP-43-positive neuronal cytoplasmic inclusions are identified in the brain and spinal cord in most cases of ALS. While TDP-43 is predominantly expressed in the nucleus, it can shuttle between the nucleus and cytoplasm and undergo various posttranslational modifications, such as phosphorylation and ubiquitination, in the cytoplasm (Tziortzouda et al., 2021). TDP-43 dysfunctions and cytoplasmic aggregation seem to be the central pathogenicity in ALS, which can result in neuronal and glial cell damage (Tamaki and Urushitani, 2022). Increased expression and activity of GSK-3β in the spinal cord, frontal, and temporal cortices have been linked to TDP-43 phosphorylation and cytoplasmic accumulation in ALS patient (Yang et al., 2008). There is a correlation between the level of GSK-3β and dysfunction in ALS individuals, indicating a potential biomarker for disease progression (Sreedharan et al., 2015). Studies have shown that treatment with a non-ATP competitive GSK-3β inhibitor, tideglusib, in TDP-43 (A315T) transgenic mice not only reduces the levels of phosphorylated TDP-43 in the mouse spinal cord but also delays symptom onset, improves motor function, and slows disease progression, further supporting the role of GSK-3β in the pathogenesis of ALS (Martínez-González et al., 2021).

### 6.2. GSK-3β regulates the Wnt signaling pathway

The Wnt signaling pathway is crucial in both physiological and pathophysiological processes of the CNS (Chen et al., 2012; Yu et al., 2013). There are two Wnt signaling pathways: the β-catenin-dependent pathway (WNT/β-catenin pathway) and the β-catenin-independent pathway, which were formerly referred to as the canonical and noncanonical Wnt signaling pathways, respectively (Akoumianakis et al., 2022). The WNT/β-catenin pathway drives neural progenitor cell differentiation into neurons, regulates hippocampal neurogenesis, enhances neural stem cell proliferation, and promotes synaptic stability and plasticity (Hayat et al., 2022). GSK-3β is a component of the complex that disrupts the WNT/β-catenin complex, phosphorylates β-catenin, and targets it for ubiquitin-proteasome degradation (Aberle et al., 1997; Duda et al., 2020). Inhibiting GSK-3β promotes the nuclear translocation of β-catenin in the lateral ventricles of postnatal mice, stimulating the proliferation of oligodendrocyte progenitor cells (Azim and Butt, 2011). Oligodendrocyte progenitor cells support myelin formation and neuronal metabolism. Therefore, inhibiting GSK-3β is considered necessary for the normal physiological function of the CNS (Lee et al., 2012). These findings support the hypothesis that GSK-3β is a crucial modulator in the pathway.

## 7. GSK-3β’s collective role in neurodegenerative diseases

Neuroinflammation is considered a contributing factor in NDDs (Gianferrara et al., 2022; Samim Khan et al., 2023). GSK-3β is a nexus for various signaling pathways, recognized as a pivotal inflammation regulator. Microglia activation and heightened proinflammatory cytokines define brain inflammation. Activated microglia release proinflammatory and neurotoxic agents, intensifying neuron harm through oxidative stress and cytokine toxicity (Kwon and Koh, 2020; Bennett and Sloan, 2021). GSK-3β activation stimulates microglia, amplifying inflammatory cytokine production, culminating in neuronal demise (Beurel, 2011; Cao et al., 2017). This perpetuates an ongoing inflammatory response, indicating GSK-3β’s vital role in the harmful feedback loop with microglia and impaired neurons. Inhibiting GSK-3β enhances tolerance to inflammation, curbing repeated cytokine release, and ameliorating symptoms in inflammatory conditions (Wang et al., 2013; Beurel et al., 2015). These findings underscore GSK-3β’s involvement in microglia-mediated neuroinflammation and neuronal death.

Mitochondria play a crucial role in the development and progression of NDD (Johnson et al., 2021). Mitochondria can synthesize ATP and regulate cellular apoptosis, ferroptosis, and inflammasome activation. Deficits in mitochondrial biogenesis may contribute to dysfunction in various neurodegenerative conditions. Drp1 is key in maintaining healthy mitochondrial dynamics by inducing fission through contraction and GTPase activity (Kleele et al., 2021). GSK-3β triggers GTPase activity by phosphorylating Drp1 at Ser40 and Ser44, resulting in fragmented mitochondria (Yan et al., 2015). Furthermore, mitochondrial dysfunction-induced hydrogen peroxide further activates GSK3β, exacerbating disease progression. Inhibition of GSK3β-induced phosphorylation can protect mitochondria, maintain energy metabolism, and rescue memory impairment in transgenic mice (Baek et al., 2017). In summary, overactive GSK-3β disrupts mitochondrial function and energy production, leading to neurodegeneration.

## 8. GSK-3β in other neurological diseases

Recent studies have also suggested potential links between GSK-3β dysregulation and other neuropsychiatric disorders, such as schizophrenia, depression, and anxiety (Cole, 2013; Tamura et al., 2016). Disrupted-in-schizophrenia 1 (DISC1) is associated with schizophrenia, and its deficiency causes behavioral abnormalities in mice (Johnstone et al., 2011). DISC1 also interacts with Translin-associated protein X (TRAXd), and GSK-3β regulates the function of DISC1/TRAXd (Weng et al., 2018). Inhibiting GSK-3β can protect DNA and restore neuronal function, thereby preserving neuron (Chien et al., 2018). Dysregulation of brain serotonin (5-HT) function is one of the pathogenic mechanisms of depression and anxiety (Carhart-Harris and Nutt, 2017). Increasing evidence has shown that GSK-3β acts as a modulator in the serotonin neurotransmission system, including direct interaction with serotonin 1B (5-HT1B) receptors in a highly selective manner and a prominent modulating effect on 5-HT1B receptor activity. Inactivation of GSK-3β in the brain by either pharmacological or genetic methods leads to amelioration of the abnormal behavior resulting from 5-HT deficiency (Beaulieu et al., 2008; Zhou et al., 2012). Therefore, targeting GSK-3β and related signaling pathways may provide therapeutic advantages for treating certain DISC1- and 5-HT-related neuropsychiatric disorders.

## 9. GSK-3β as a therapeutic target

GSK-3β plays a crucial role in many neurological disorders. GSK-3β may serve as a promising therapeutic target for treating diseases, promoting recovery, and maintaining organism stability (Rippin and Eldar-Finkelman, 2021). Several categories of GSK-3β inhibitors have been developed (Wadhwa et al., 2020), including (1) magnesium-competitive inhibitors; (2) ATP-competitive inhibitors; (3) substrate-competitive inhibitors; and (4) regulators of GSK-3β Ser9 phosphorylation.

The currently used drugs for AD, including cholinesterase inhibitors and NMDA receptor antagonists, only mildly alleviate the symptoms but do not halt or delay the progression of the disease (Birks and Harvey, 2018; McShane et al., 2019). Lithium salts are one of the earliest drugs used for AD treatment and can inhibit GSK-3β and reduce Tau phosphorylation and amyloid production but they have poor efficacy for mild AD patient (Hampel et al., 2009). The selective inhibitors SB216763 and FLZs can reduce Tau phosphorylation and protect neurons from the toxicity of Aβ oligomers, improving spatial memory (Bao et al., 2013; Deng et al., 2014). Noncompetitive irreversible Adenosine Triphosphate (ATP) inhibitor derivatives of 4-benzyl-2-methyl-1,2,4-thiadiazolidine-3,5-dione (TDZD), such as Tideglusib and TDZD-8, can stimulate GSK-3β Ser9 phosphorylation, thereby reducing Tau and amyloid deposition (Domínguez et al., 2012). Although there are many potential inhibitors available for AD treatment, few have succeeded in clinical trials (Chauhan et al., 2022).

GSK-3β inhibitors, including SB216763, lithium chloride, and TDZD-8, have demonstrated significant effects in the treatment of PD. These inhibitors efficiently inhibit the generation of proinflammatory cytokines such as TNF-α and IL-12 while promoting the production of anti-inflammatory cytokines such as IL-10 (Coant et al., 2011), which helps protect dopaminergic neurons from neurotoxicity, preserve mitochondrial function, and eliminate oxidative damage in PD model (Chiu et al., 2014). Additionally, GSK-3β inhibitors can promote the proliferation of neural progenitor cells and stimulate neurogenesis, playing a role in neural regeneration and repair (Eom and Jope, 2009). Therefore, GSK-3β inhibitors have broad application prospects in the treatment of PD.

GSK-3β inhibition has emerged as a potential therapeutic approach for HD (D'Mello, 2021). Studies have revealed GSK-3β’s association with mHtt aggregation in HD mice and neurons (Valencia et al., 2010). Elevated GSK-3β expression and activity in the hippocampus of HD patients and mouse models correlate with increased tau phosphorylation (L'Episcopo et al., 2016). In cellular and mouse HD models, GSK-3β silencing and inhibition reduce mutant huntingtin aggregates and neuronal death, while selective GSK-3 inhibition improves motor function and neuroprotection (Rippin et al., 2021). Lithium, a GSK-3β inhibitor, exhibits beneficial effects in preclinical HD models, enhancing motor function and mitigating striatal deficits (Snitow et al., 2021). Co-treatment with lithium and valproate further alleviates HD-associated deficits (Chiu et al., 2011). These findings underscore GSK-3β’s relevance in HD pathogenesis, driving ongoing exploration of its therapeutic potential.

GSK-3β inhibitors have been studied as potential therapies for ALS *in vivo*. It has been shown that lithium treatment can prevent apoptosis, alleviate motor function defects, and slow disease progression in SOD1-G93A mice (Shin et al., 2007). Another potential therapeutic option for ALS is valproic acid (VPA), which has been shown to slow disease progression and increase lifespan in SOD1-G93A mice (Sugai et al., 2004). Combined therapy with lithium and VPA shows greater rescue effects on motor dysfunction and disease progression in the SOD1-G93A mouse model (Jiang et al., 2016). These findings indicate that GSK-3β holds great potential as a therapeutic target for ALS.

## 10. Conclusion

NDDs such as AD, HD, PD, and ALS are increasingly recognized as major contributors to death and disability worldwide (Carroll, 2019). Although these four common NDDs exhibit different clinical profiles, they share common molecular pathogenic mechanisms. These mechanisms encompass proteostasis, cellular signaling pathways, neuroinflammation, and mitochondrial deficits, suggesting converging pathways of neurodegeneration (Durrenberger et al., 2015; Noori et al., 2021). GSK-3β plays a pivotal role in multiple NDDs. In AD, GSK-3β influences the processing of APP and hyperphosphorylation of Tau protein, contributing to Aβ plaque formation and neurofibrillary tangle development. In PD, GSK-3β affects α-synuclein aggregation and dopamine receptor signaling. In HD, GSK-3β contributes to Tau pathology and GABAergic dysfunction. In ALS, GSK-3β impacts TDP-43 pathology and the Wnt signaling pathway. The intricate involvement of GSK-3β in the pathogenesis of various NDDs underscores its significance as a potential therapeutic target. GSK-3β inhibitors have the potential to protect neurons in various disease models. In conclusion, by unraveling its diverse functions and their interactions, we can advance our understanding of disease mechanisms and design targeted strategies to counteract neurodegeneration.

## 11. Discussion and future directions

The diverse functions of GSK-3β in controlling protein aggregation, mitochondrial dysfunction, neuroinflammation, and cellular signaling contribute to a comprehensive understanding of disease mechanisms. Although GSK-3β’s involvements in AD, PD, HD, and ALS are established, its potential impact on other neurodegenerative and neuropsychiatric disorders presents an intriguing field for further investigation (Lin et al., 2020).

The interactions of GSK-3β with the Wnt pathway, dopamine receptor signaling, and various kinases are intricately complex. Delving into these interactions deepens our understanding of potential molecular mechanisms and may unveil novel treatment strategies. Selectively modulating the activity of specific cellular pools of GSK-3β or its specific downstream or upstream partners may offer effective approaches for combating neurodegenerative diseases. Thus, GSK3β and its signaling pathway partners hold great promise as therapeutic targets for a multitude of neurological disorders.

A global endeavor is currently in progress to discover more effective methods for clinically managing NDDs, aiming to delay their onset and hinder their progression. Inhibition of GSK-3β was considered a promising therapeutic approach. However, most GSK-3β inhibitors that were developed function as ATP competitive inhibitors, with typical limitations in specificity, safety, and drug-induced resistance. Clinical and preclinical trials with GSK-3β inhibitors have shown limited effectiveness (Llorens-Martín et al., 2014; Xia et al., 2021). Thus, more selective and potent GSK-3β inhibitors should be developed, which can effectively target pathological processes and spare essential physiological functions. Additionally, considering the complex nature of NDDs, it appears that single-target drugs might fall short of achieving satisfactory therapeutic outcomes. Multi-target design strategy may potentially enhance both therapeutic safety and efficacy (Zhang et al., 2019; Gontijo et al., 2020).

## Author contributions

HY wrote the draft. MX revised the manuscript. ZZ conceived the manuscript and made revisions. All authors contributed to the article and approved the submission.

## Funding

This work was supported by grants from the Innovative Research Groups of Hubei Province (2022CFA026) and the National Natural Science Foundation of China (Nos. 82271447 and 81901090).

## Conflict of interest

The authors declare that the research was conducted in the absence of any commercial or financial relationships that could be construed as a potential conflict of interest.

## Publisher’s note

All claims expressed in this article are solely those of the authors and do not necessarily represent those of their affiliated organizations, or those of the publisher, the editors and the reviewers. Any product that may be evaluated in this article, or claim that may be made by its manufacturer, is not guaranteed or endorsed by the publisher.
